# Analysis and prediction of protective continuous B-cell epitopes on pathogen proteins

**DOI:** 10.1186/1745-7580-4-1

**Published:** 2008-01-07

**Authors:** Johannes Sollner, Rainer Grohmann, Ronald Rapberger, Paul Perco, Arno Lukas, Bernd Mayer

**Affiliations:** 1 emergentec biodevelopment GmbH, Rathausstrasse 5/3, A-1010 Vienna, Austria; 2 University of Vienna, Institute for Theoretical Chemistry, Dr. Bohrgasse 6, A-1030 Vienna, Austria; 3 Medical University of Vienna, Department of Nephrology, Währingergürtel 18-20, A-1090 Vienna, Austria

## Abstract

**Background:**

The application of peptide based diagnostics and therapeutics mimicking part of protein antigen is experiencing renewed interest. So far selection and design rationale for such peptides is usually driven by T-cell epitope prediction, available experimental and modelled 3D structure, B-cell epitope predictions such as hydrophilicity plots or experience. If no structure is available the rational selection of peptides for the production of functionally altering or neutralizing antibodies is practically impossible. Specifically if many alternative antigens are available the reduction of required synthesized peptides until one successful candidate is found is of central technical interest. We have investigated the integration of B-cell epitope prediction with the variability of antigen and the conservation of patterns for post-translational modification (PTM) prediction to improve over state of the art in the field. In particular the application of machine-learning methods shows promising results.

**Results:**

We find that protein regions leading to the production of functionally altering antibodies are often characterized by a distinct increase in the cumulative sum of three presented parameters. Furthermore the concept to maximize antigenicity, minimize variability and minimize the likelihood of post-translational modification for the identification of relevant sites leads to biologically interesting observations. Primarily, for about 50% of antigen the approach works well with individual area under the ROC curve (AROC) values of at least 0.65. On the other hand a significant portion reveals equivalently low AROC values of < = 0.35 indicating an overall non-Gaussian distribution. While about a third of 57 antigens are seemingly intangible by our approach our results suggest the existence of at least two distinct classes of bioinformatically detectable epitopes which should be predicted separately. As a side effect of our study we present a hand curated dataset for the validation of protectivity classification. Based on this dataset machine-learning methods further improve predictive power to a class separation in an equilibrated dataset of up to 83%.

**Conclusion:**

We present a computational method to automatically select and rank peptides for the stimulation of potentially protective or otherwise functionally altering antibodies. It can be shown that integration of variability, post-translational modification pattern conservation and B-cell antigenicity improve rational selection over random guessing. Probably more important, we find that for about 50% of antigen the approach works substantially better than for the overall dataset of 57 proteins. Essentially as a side effect our method optimizes for presumably best applicable peptides as they tend to be likely unmodified and as invariable as possible which is answering needs in diagnosis and treatment of pathogen infection. In addition we show the potential for further improvement by the application of machine-learning methods, in particular Random Forests.

## Background

The applicability of peptides for the generation of preventive vaccines, therapeutics and diagnostics is an actively investigated field. Although historically disfavored the application of peptides in vaccine design is currently experiencing a renaissance[1]. While the focus is often on T-cell responses especially the generation of B-cell responses is of relevance against certain pathogens such as HIV to prevent initial infection [2]. It is thus not surprising that since the early days of computational biology scientists have attempted to predict the relevance of protein domains and peptides in several areas of application. Initial hallmarks of the field are represented, among many others, by work of Hopp and Woods [3,4]. During the following and more recent years various methods and problems concerning the prediction of continuous B-cell epitopes have been proposed [5-12]. Recently the usability of amino acid scales for the prediction of B-cell epitopes has been profoundly questioned [13] and common standards regarding the validation of epitope predictions have been discussed [14].

Generally, B-cell antigenicity predictions should probably be understood as a measure of the likelihood to develop antibodies against a particular determinant or part of a surface, rather than another. In addition, most proposed classifiers of continuous epitopes are ultimately a composite of accessibility and charge-interaction potential prediction with a strong focus on delivering a few experimentally applicable peptides rather than an overall complete probability distribution for raising antibodies. In addition, continuous epitopes make up an undefined but presumably small part of the complete "epitope space" of an antigen. This even so when assuming distinct epitopes rather than a continuous surface and accepting dominant continuous elements of structural epitopes as continuous epitopes.

In this study we extend previous work by investigating a subgroup of continuous B-cell epitopes, namely protective continuous epitopes. This aspect has to the best of our knowledge not been systematically tackled so far.

From a biological point of view several principles should govern the availability and evolutionary behavior of protective amino-acid sites on proteins. One of the questions we ask is whether these principles or constraints lead to signals which can be used for predicting or rather detecting candidate epitopes. We assume that an antibody (and hence it's epitope) is protective because the function of the antigen (target protein) is inhibited and the activity of the organism is thus reduced. Or alternatively because immunological processes are activated leading to the destruction of the organism. The prior (protectivity class I) might most likely be expected in adhesion molecules or pathogenicity factors like matrix degrading proteases and toxins.

The second category (protectivity class II) would primarily refer to downstream events of antibody induced complement activation such as pore formation and opsonization leading to phagocytosis. It can be considered likely that the two mechanisms would often lead to differently characterized epitopes. Consequently as different functional constraints can be expected separate strategies for detecting them may be required. Basically any protein of high expression and density on the surface with at least one good epitope can be the target of class II protectivity. We define a "good" epitope as a surface area of high interaction potential, shape complementarity to the basic layout of an antibody [15] and dissimilar to self. Predicting that class therefore also requires to assess (or estimate) the density of a protein on the cell during pathogenesis, optimally experimentally or by inference from related organisms. A practically applicable continuous epitope could then be any exposed, possibly evolutionarily highly variable loop with an amenable antigenicity and solvent-accessibility profile. Class II protectivity may often be comparably straightforward to predict as soon as a target protein has been identified because selection of high scoring B-cell epitope scores often seems to be relatively straightforward and selection routines primarily falter in the domain of suboptimal scores as has been indicated by Sollner and Mayer [9]. However, immunologically sub-dominant or even cryptic B-cell epitopes can be of special interest regarding protectivity and inter-strain cross-reactivity [16] and are sometimes consistently immuno-silent during natural infection [17,18]. As a consequence we focus on conserved and therefore presumably functionally constrained epitopes without post-translational modifications which still exhibit amenable antigenicity scores. Regarding the previous definition these epitopes may often fall into class I of protective continuous epitopes.

Such principles are primarily valid for pathogens already adapted to the host. Organisms in the process to adapt to new hosts or receptors can undergo significant alterations in their antigenic structure as has been demonstrated for SARS virus [19-21] and HIV [22], respectively.

We speculate that by means of functional constraints centers of biological activity can exert conserving pressure on closely associated potential epitopes while less relevant regions can be more variable. It may also be viable to suggest that conservation of posttranslational modification patterns may be different when comparing highly variable exposed loops and sites of functional relevance as modifications can play a major role in the masking of protective epitopes [23,24] but may often be undesired near functional centers.

As Class II protectivity is to a certain degree already approached by standard B-cell epitope prediction (the maximization of antigenicity) and on the other hand depends on a bioinformatically more elusive factor (expression levels of pathogen protein) we see reason to focus on the prediction of conserved, functionally constrained epitopes (class I protectivity).

This work assesses in how far correlation between antigenicity, variability, post-translational modifications and protectivity/functional relevance can be put to use in a predictive model without the availability of 3D data. To compensate for the lack of 3D data multiple alignments of selected proteins are harnessed to derive information regarding the conservation of post-translational modification motifs as well as sequence variability per-se. We believe that understanding evolutionary "movement" of pathogen proteins allows insights into the importance of potential epitopes and we interpret such importance as indicator of protectivity.

## Results

**Figure 1:**
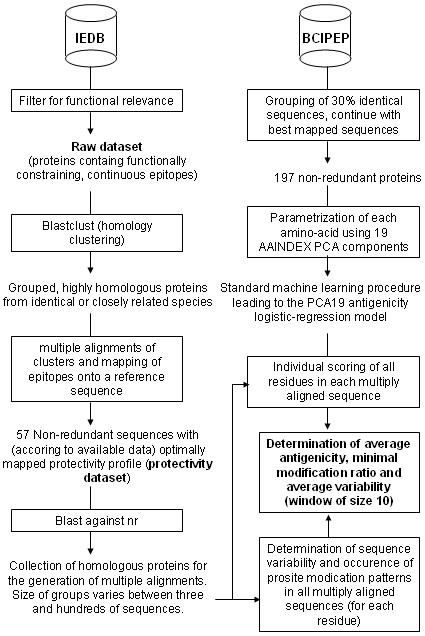
The figure shows the first part of the overall workflow applied in this project.

**Figure 2:**
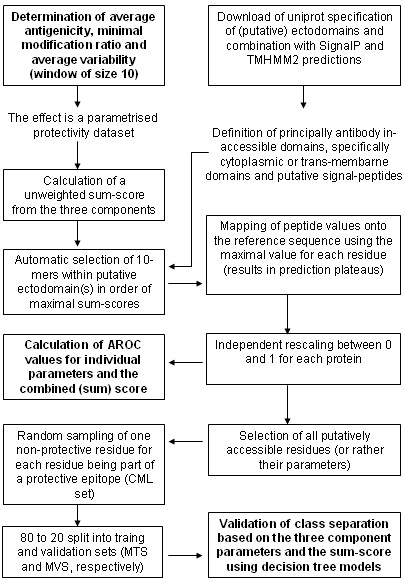
The figure shows the second part of the overall workflow applied in this project.

Classification into presumably protective or non-protective epitopes is conducted using three independently determined parameters: predicted B-cell antigenicity, sequence variability and conservation of post-translational modification motifs. As described in the *Methods *section used antigenicity, variability and motif-conservation scores are based on multiple-alignments i.e. each sequence contributes to a composite value. All values are determined within 10-mers which slide over the alignments and overlap by 9 amino-acids. B-cell antigenicity and sequence variability are averaged within these 10-mers. We chose this size to use an intermediate between common assumptions about sizes of continuous epitopes (usually between 7 and 15 amino-acids). The maximum ratio of post-translational modifications over all constituent alignment columns is used within the same area. In other words, for the prior two the 10-mer values are calculated as the averages over the averages calculated from individual alignment columns. The latter seeks the maximal ratio of possibly modified amino-acids over all alignment-columns in the area because it presumably is most indicative of actual modifications. An overview over the major steps in the workflow has been highlighted in figures 1 and 2.

### Antigenicity Classification

#### Logistic regression models based on PCA of 505 amino acid propensity scales

We examined regression models based on 505 amino acid propensity scales. As described in *Methods *each of these scales characterizes amino acid residues regarding specific properties by assigning a value. From this representation, the information of 505 amino acid propensity scales was transformed into 19 principal components applying principal component analysis.

Based on these 19 principal components, a logistic regression model was derived. It is in the following mentioned as PCA19.

Briefly, a dataset of 197 proteins was obtained by clustering all BCIPEP sequences with at least 30% identity. This step removed redundancies potentially biasing validation procedures. In a second step non-antigenic amino-acids were randomized (maintaining the original amino-acid composition) to avoid the misclassification of unknown epitopes.

Each amino-acid of those proteins was then parameterised using all of the described 19 components. Validation on the training set by bootstrap analysis in combination with a logistic regression indicated an AROC value of 0.60.

#### Validation of antigenicity predictions on an independent dataset

To obtain an unbiased impression of the performance of the PCA19 classifier compared to an accepted gold standard such as ABCpred [25] an independent validation-dataset published by Blythe and Flower was used. To make methods compatible ABCpred predictions were run with standard settings except that the threshold was lowered to 0.1. Scores reported for peptides by ABCpred were assigned to each comprising amino-acid where larger values superseded the prediction of an overlapping peptide. AROC values were calculated. Both methods performed close to random (as is not too astonishing concerning the findings by Blythe et.al), with AROC values of 0.55 and 0.52 for PCA19 and ABCpred, respectively. These results are relativated later in this work when using only potentially relevant domains of a protein antigen, indicating systematic problems of the way B-cell epitope prediction validation is usually conducted.

### Protectivity Analysis and Prediction

#### Effect of domain-accessibility filtering on protectivity prediction

To assess the effect of domain accessibility filtering (masking) from protectivity prediction AROC values of the described linear parameter combination before and after filtering were compared. Whereas the median AROC over all protein was determined as 0.56 before masking of presumably inaccessible trans-membrane or cytoplasmic domains it increased to 0.65 afterwards. While the AROC before masking is comparable to the one obtained by antigenicity prediction on the Blythe and Flower validation dataset the improvement to 0.65 strongly indicates the benefit of the procedure. Domain accessibility filtering can be considered an aspect of fair evaluation in B-cell epitope classification as a whole as it can be assumed that continuous epitopes of accessible domains are more likely mapped or otherwise reported than others, besides the protectivity aspect. While it may be argued that inclusion of Uniprot data into the process adds an aspect of human intervention we see that many data-sources can and are used for the annotation of putative ectodomains. Among those are also experimental data, which is in itself not a problem for bioinformatical validation strategies as long as the validation dataset was not engineered to fit these data particularly well. That is not the case. The domain filter was simply built by manually collecting different data-sources according to simple rules as we considered automated harvesting for 57 proteins and unnecessary effort.

#### Prediction of protective linear epitopes using a sum-score

To evaluate the prediction of protective linear epitopes a new validation dataset was generated as described in Methods and Data. Briefly, the IEDB resource was queried for pathogen proteins with linear antibody determinants which lead to a biological effect upon interaction.

In viral polyproteins commonly only the dominant surface proteins were used as could be expected for a newly sequenced pathogen without in detail knowledge. To limit predictions to candidate regions (i.e. possibly immunologically accessible regions) domain accessibility filtering was applied. To do so domain data regarding polyproteins, trans-membrane structures or signal-peptides (predicted or experimentally determined) were taken from UniProt [26] or predicted using the TMHMM v.2.0 [27] and SignalP 3.0 Servers [28]. Domains which were not considered relevant for protective B-cell responses were intracellular domains of trans-membrane proteins, the first 10 amino-acids of a putative extra-cellular domain after a trans-membrane region and leader-peptides. Briefly, proteins were completely scored for antigenicity/protectivity but amino-acid scores in regions outside domains assumed to be surface exposed were set to 0 thus leading to a generic classification as non-protective. Masked amino-acid stretches were still considered for ROC calculation to reflect the impact of the analysis as a whole. AROC measures were thus based on completely scored proteins partially set to 0.

For each amino-acid of proteins in the protectivity dataset variability and percentage of modifications (ratio of sequences which carry a modification motif indicating this specific amino acid versus all aligned sequences) based on multiple alignments were calculated as described earlier. Finally the average predicted antigenicity was calculated for each alignment column. Each of the three sub-scores was then rescaled between 0 and 1 for easier comparability.

To asses the power of score-combinations antigenicity, 1 – variability and 1 – PTM ratio were summed for all overlapping 10-mers where the score was assigned to the central amino-acid.

See Table 1 for AROC values of individual proteins using any of the three sub-scores alone as well as the linear combination (sum score). The last row of the table indicates the overall AROC when analyzing all unmasked amino-acids together.

**Table 1:** protectivity AROC values for individual proteins separately derived from predictions based either on antigenicity, 1 – modifications or 1 – variability as well as a combined (sum) score for individual proteins

**protein function**	**protein**	**antigenicity**	**modifications**	**variability**	**combined**
attachment/fusion	P11224	0,51	0,15	0,42	0,15
attachment/fusion	2327073	0,77	0,69	0,77	0,77
attachment/fusion	1930067	0,83	0,51	0,64	0,65
attachment/fusion	P09592	0,66	0,77	0,57	0,74
RNA encapsidation	13559809	0,62	0,48	0,50	0,46
enzyme/secreted cytolysin	P13128	0,45	0,47	0,43	0,45
RNA encapsidation	37724690	0,29	0,74	0,26	0,67
toxin/translocation/binary-toxin	Q46221	0,42	0,28	0,10	0,11
attachment/capsid	P30129	0,77	0,35	0,07	0,29
DNA replication	138881	0,31	0,31	0,14	0,13
toxin	P01558	0,41	0,49	0,29	0,41
host evasion/IgG binding	13622466	0,88	0,61	0,86	0,76
attachment/fusion	116774	0,60	0,40	0,26	0,41
**enzyme/host evasion**	**15644702**	**0,68**	**0,55**	**0,65**	**0,63**
enzyme/protease	P10845	0,91	0,55	0,60	0,77
**attachment/fusion**	**P03449**	**0,59**	**0,50**	**0,45**	**0,49**
**transport**	**P13794**	**0,82**	**0,75**	**0,64**	**0,81**
attachment/fusion	P08669	0,63	0,58	0,67	0,70
attachment (?)	Q02938	0,82	0,60	0,54	0,73
enzyme/protease/tissue invasion	30260755	0,18	0,80	0,94	0,85
**unknown function**	**P13403**	**0,55**	**0,75**	**0,51**	**0,73**
unknown function	P13664	0,46	0,68	0,91	0,70
unknown function	42374894	0,62	0,58	0,64	0,66
toxin/translocation/binary-toxin	P13423	0,59	0,62	0,22	0,56
unknown function	14162008	0,23	0,23	0,11	0,08
attachment/fusion	P59594	0,57	0,45	0,59	0,52
**transport**	**P16567**	**0,47**	**0,51**	**0,20**	**0,31**
unknown function	13621499	0,35	0,30	0,65	0,36
attachment (?)	13622014	0,27	0,46	0,16	0,39
unknown function	15675130	0,64	0,96	0,73	0,92
unknown function	13622584	0,63	0,56	0,72	0,65
unknown function	790646	0,98	0,83	0,25	0,95
host evasion/resistance to phagocytosis (?)	P26948	0,62	0,57	0,61	0,70
unknown function	P21206	0,54	0,52	0,43	0,58
enzyme	153640	0,35	0,88	0,80	0,87
transport	13623184	0,55	0,70	0,65	0,69
attachment/fusion	P35253	0,56	0,45	0,27	0,38
host evasion/resistance to phagocytosis/toxin	P55128	1,00	0,40	0,54	0,73
**attachment/fusion**	**P03524**	**0,54**	**0,73**	**0,65**	**0,73**
host evasion/superantigen	P06886	0,90	0,68	0,37	0,80
toxin	P0A0L2	0,43	0,54	0,49	0,46
**host evasion/superantigen**	**P01552**	**0,34**	**0,59**	**0,38**	**0,53**
attachment	97812	0,80	0,57	0,80	0,73
attachment/fusion	P27662	0,66	0,43	0,72	0,50
**host evasion/resistance to phagocytosis**	**P12379**	**0,44**	**0,47**	**0,35**	**0,41**
**host evasion/resistance to phagocytosis**	**P02977**	**0,32**	**0,37**	**0,25**	**0,33**
transport	13621681	0,64	0,41	0,54	0,46
toxin/peptidase/binary-toxin	P15917	0,51	0,22	0,65	0,21
attachment/fusion	P33478	0,71	0,61	0,69	0,69
**unknown function**	**P19597**	**0,85**	**0,87**	**0,61**	**0,86**
attachment/fusion	P07946	0,71	0,46	0,31	0,53
attachment/fusion	Q05320	0,43	0,54	0,18	0,38
attachment/RNA encapsidation	P03308	0,88	0,86	0,71	0,82
attachment/fusion/neuraminidase	O89343	0,19	0,88	0,31	0,71
attachment/fusion	P05769	0,93	0,97	0,91	0,97
attachment/RNA encapsidation	P08617	0,86	0,82	0,86	0,90
**attachment/fusion**	**Q69091**	**0,89**	**0,69**	**0,45**	**0,78**
*merged (contaminated)*		*0,66*	*0,62*	*0,62*	*0,65*
*mean non-contaminated*		*0,60*	*0,56*	*0,52*	*0,59*
*mean contaminated*		*0,60*	*0,57*	*0,51*	*0,59*
*median non-contaminated*		*0,62*	*0,56*	*0,56*	*0,65*
*median contaminated*		*0,55*	*0,59*	*0,51*	*0,63*

Proteins highlighted in bold are markedly homologous or identical to sequences used for the training of the PCA19 antigenicity classifier. Values derived from those proteins are denoted as "contaminated". The last five rows compare overall (global) classification performances of concatenated (merged) proteins as well as mean and median values between individual parameters and the combined score. Note that "merged" does not mean averaged and is thus biased by the length of individual proteins.

**Figure 3:**
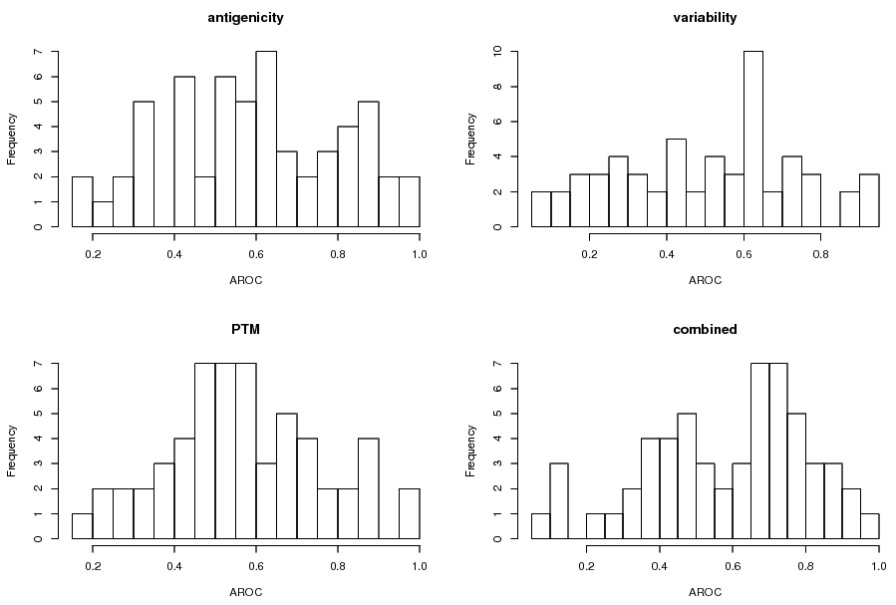
The figure shows the distribution of AROC values for protectivity predictions solely based on antigenicity, PTM (post translational modifications), variability as well as the sum score of the three.

Considering the AROC merged value (resulting from the concatenation of all putatively accessible domains after scoring) antigenicity alone outperforms all other scores, including the combined one. Yet, although Table 1 indicates overall better performance of antigenicity, the distribution of AROC values for individual proteins indicates a different view. Table 2 shows that for the combined score fewer proteins fall in the very low AROC area (7 versus 10 are < = 0.35) whereas substantially more (30 versus 21) fall in the AROC area we considered good (> = 0.65). Eight of the 57 proteins used for protectivity prediction are similar or identical to sequences used for training the PCA19 classifier (as identified using blastp). To evaluate this bias/contamination the AROC value medians of truly independent versus dependent (contaminated) proteins has been listed in Table 2. Interestingly and against expectation contaminated proteins underperform when measured by median compared to independent entries leading to the observation that no pre-emptive separation of shared sequences is necessary in this case. For an overview of AROC distributions see the histograms in figure 3.

**Table 2:** The table lists AROC performance of protectivity classifications for individual proteins based on different parameters

**AROC**	**antigenicity**	**modification**	**variability**	**combined**
**proteins where AROC < = 0.35**	10	7	16	7
**proteins where AROC > = 0.65**	21	19	16	30
**AROC median non-contaminated**	0.62	0.56	0.56	0.65
**AROC median contaminated **	0.55	0.59	0.45	0.63

Note that measured by the AROC median non-contaminated proteins unexpectedly seem to perform better than contaminated sequences. The combined classifier yields higher median AROC values than classifications based on individual parameters. Possibly more important, the number of proteins with AROC values > = 0.65 is substantially higher when comparing the combined score (protein count 30) with the best single score antigenicity (protein count 21).

Taken together 25 or 30 (44 or 53%) of 57 investigated proteins reveal AROC values > = 0.65 depending on whether contaminated proteins are neglected or not. This population is characterised by mean and median AROC values of 0.77 and 0.74, respectively. We interpret this as a roughly 50% probability that protectivity prediction will significantly enhance selection of peptides for the stimulation of protective immune responses for a particular protein, given that a relevant protein has been identified beforehand. We also want to point out that overall up to 65% of proteins show AROC values < = 0.35 or > = 0.65.

#### Synthesis Score

As described in *Methods *a "Synthesis Score" representing the number of peptides required for likely experimental success is a relevant readout concerning minimization of experimental cost. For vaccine design it is crucial to limit the number of synthesized peptides which enter experimental validation to a practically feasible amount. How many peptides can be synthesized depends on budget and resources as well as ethical considerations regarding the number of lab-animals, entities which are tied to the number of proteins (antigens) to be screened as well as available time. To provide such a measure we defined the size of peptides to be synthesized as 17-mers (a size commonly used by us) and the minimal overlap with protective epitopes to call it a hit as five amino-acids. Selected peptides could overlap, but each new epitope had to be centered on a hitherto uncovered amino-acid. These central amino acids were selected by maximizing the described sum score of antigenicity + (1-variability) and (1-maximal modification ratio).

Following this procedure we determined how many peptides had to be selected per protein to provide a likely working selection for at least 50% of screened proteins. The presented combination method required six peptides to be selected compared to eight for random picking. This compares to five versus eight peptides when only the 30 proteins with AROC > = 0.65 were looked at. Note that random picking of central-amino acids was also restricted to the regions not filtered out by domain-exclusion to warrant fairness in the comparison.

By synthesizing and validating a maximum of six peptides, on average every second potentially protective protein can be expected to be the source of likely quite conserved, optimally unmodified, continuous and protective or at least functionally altering epitopes.

#### Analysis of feature correlation in protective epitopes

To assess the relationship of antigenicity, variability and modifications Pearson correlation-coefficients were calculated. After domain-filtering all distinct (non-overlapping) protective regions were analyzed separately to obtain an idea whether a common trend could be identified. In summary, overall average and median correlation were not significant between antigenicity, variability and modification ratio (generally < = 0.30 and > = -0.18). For each combination a subset of epitopes showed high correlation, however. Results have been summarized in Table 3.

**Table 3:** The table lists numbers of protective epitopes exhibiting negative or positive feature association when considering all six possible combinations of antigenicity, modification percentage, variability and evolutionary constraint index (ECI)

	**antigenicity**	**modification**	**variability**	**ECI**
**antigenicity**	0/110	33/14	31/26	26/23
**modification**	-	0/110	22/27	19/21
**variability**	-	-	0/110	15/31
**ec**	-	-	-	0/110

Note that like for the prediction of protectivity the used modification and variability scores (features) have been used as 1-value, so actual associations have to inverted as well. Each field in the table contains a pair of numbers where the first and second indicate the number of epitopes associated with a Pearson coefficient of < = -0.5 and > = 0.5, respectively. High numbers therefore indicate a strong degree of feature association.

Note that no pair of features shows significant positive or negative correlation for more than 30% of the total number of 110 considered epitopic regions. However, for all pairs of sub-scores strong positive and negative associations exist. In the case of antigenicity and variability more than 50% of analyzed epitopes show strong correlation between the two, but at practically equal numbers positive and negative association. This means that roughly 25% of epitopes are either markedly antigenic and conserved or non-antigenic and variable. Furthermore another 25% are markedly antigenic and variable or non-antigenic and conserved. Protective epitopes seem to be tendencially positively correlated considering antigenicity and conservation of motifs for posttranslational modification while variability and the evolutionary constraint index (ECI) are often negatively associated, as could be expected. Unfortunately no association between protein functional class and type of correlation could be detected (data not shown). Preliminary experiments evaluating the potential power of the correlation coefficient as a new parameter for protectivity prediction indicated close to random performance (data not shown).

The previously described observation that for about 65% of proteins either substantially good or bad predictive power can be seen suggests that the described 30% correlated epitopes are distinct sets depending on the parameter-combination. Alternatively it may also be that weaker correlations than -0.5 and +0.5 can still positively influence AROC.

#### Machine Learning models

An unweighted linear combination score is a simple way to combine parameters without optimizing a model, i.e. as a first approach strategy or if non independent optimization dataset exists. To extend this strategy, albeit without the option to analyse the unbiased effect on the entire protectivity dataset, machine-learning procedures were applied.

In particular, to analyse the validity of using all three parameters as well as the relative merit of the sum-score the protectivity dataset was converted into a format amenable for machine learning procedures such as decision trees. As described in the *Methods *section the entire protectivity set of 57 proteins was subjected to domain filtering to restrict to possibly accessible residues which were then compiled into the CML set. The class separation baseline of this set is 93,01%, due to the massive domination of non-protective residues. Using WEKA standard settings a C4.5 decision tree achieved 96.12% separation on the same set.

**Figure 4:**
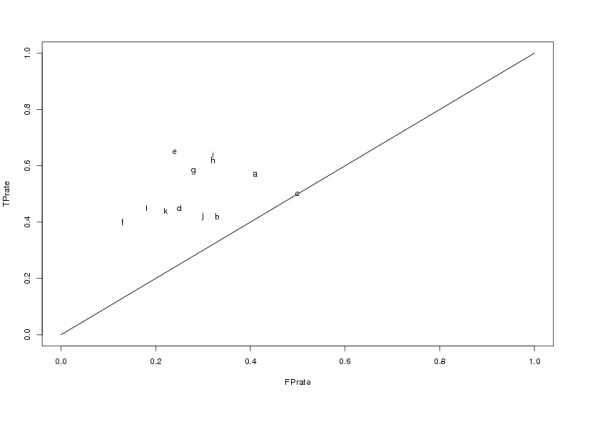
The figure shows the ROC plot of C4.5 decision trees used to determine the relevance of individual parameters and parameter combinations for the prediction of protectivity. For details on the obtained classifications please see table 4.

Because relative merits of machine learning are difficult to assess on strongly biased datasets such as CML, balanced and stratified training and validation sets (MTS and MVS, respectively) were randomly sampled from CML to make independent validation possible. Using the training set (MTS) and again applying C4.5 different parameter combinations were assessed. Results can be seen in table 4 and figure 4. The decision tree algorithm was applied because for single parameters this should basically be a search for the entropically optimal class-split whereas for parameter combinations also non-linear relationships (particularly the presence of distinct groups) can be captured. The table shows that each parameter contributes significantly and the best model comprises all three attributes as well as the sum-score as a fourth attribute. Independent of variability alone shows the best class-separation among individual parameters. Interestingly PTM alone yields no improvement over random classification while the combination with the remaining features significantly boots its impact.

**Table 4:** The table shows the class separation measured by 10-fold stratified cross-validation using a C4.5 decision tree learner on the training dataset (MTS). In each row a different combination of input parameters was used as indicated by X (present) in the last four columns. Separation baseline for this dataset is 50%

Nr	%sep	TN	FP	FN	TP	FPrate	TPrate	score	antigen	PTM	var
a	57.78	1086	759	799	1046	0.41	0.57	**X**	**-**	**-**	**-**
b	54.58	1242	603	1073	772	0.33	0.42	**-**	**X**	**-**	**-**
c	49.86	920	925	925	920	0.50	0.50	**-**	**-**	**X**	**-**
d	60.08	1382	463	1010	835	0.25	0.45	**-**	**-**	**-**	**X**
e	70.41	1399	446	646	1199	0.24	0.65	**X**	**X**	**X**	**X**
f	63.47	1607	238	1110	735	0.13	0.40	**-**	**X**	**X**	**X**
g	65.42	1337	508	768	1077	0.28	0.58	**X**	**-**	**X**	**X**
h	65.28	1259	586	695	1150	0.32	0.62	**X**	**X**	**-**	**X**
i	66.18	1263	582	666	1179	0.32	0.64	**X**	**X**	**X**	**-**
j	56.02	1298	547	1076	769	0.30	0.42	**-**	**X**	**X**	**-**
k	61.00	1446	399	1041	804	0.22	0.44	**-**	**X**	**-**	**X**
l	63.82	1520	325	1010	835	0.18	0.45	**-**	**-**	**X**	**X**

Column abbreviations: %sep (% class separation), score (sum score), antigen (PCA19 derived antigenicity score), PTM (post-translational modifications), var (variability score), TPrate (True Positive rate) and FPrate (False Positive rate).

To assess the performance of a somewhat more sophisticated machine-learning technique validation using the validation set and cross-validation are compared in table 5 for a C4.5 tree and a Random Forest. Obviously there is much potential for improvement over a single decision tree as the Random Forest achieves a class separation power of up to 83–84% compared to 70–73% using a single tree.

**Table 5:** The table compares the performance of a decision tree derived using the C4.5 algorithm and a Random Forest, both generated with standard parameters. Ten-fold stratified cross-validation (on MTS) and validation on an independent protectivity validation set (MVS) are compared

	C4.5			Random Forest		
	% sep	FPrate	TPrate	% sep	FPrate	TPrate

Cross-validation	70.41	0.24	0.65	83.39	0.15	0.82
Validation-set	73.64	0.26	0.73	84.27	0.15	0.83

Column abbreviations: %sep (% class separation), FPrate (False Positive rate) and TPrate (True Positive rate).

Analysis of the C4.5 tree generated from the validation set indicates the merit of all four attributes including the sum-score as an additional parameter as all four were incorporated by the algorithm (tree not shown). In addition the resulting tree is astonishingly complicated (109 leaves) when considering that only four parameters were used, again indicating the existence of different clusters. Preliminarily, these groups may well be interpreted as distinct epitope signatures pointing towards different types of protective epitopes. In other words, different but recognizable epitope profiles should be considered. It also becomes obvious that a single unweighted combination score has its merit but is outperformed by variability alone and both of them by decision tree based multi-parameter models, at least in the stratified data representation. Also, the sum-score proves to be an important additional component in the decision models as can be seen by the increase from 63% class separation to 70% class separation by inclusion of this extra feature.

#### Exemplary prediction of a protective continuous epitope

**Figure 5:**
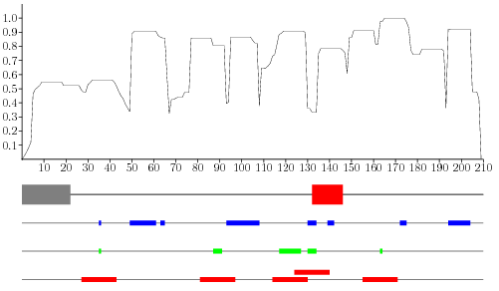
The figure plots the classification results of the C4.5 tree (in blue), the Random Forest (in green) as obtained during validation in comparison to the signal peptide (fat grey bar) and the published continuous epitope (fat red bar). On top the values for the sum-score are plotted. The last line indicates the hypothetical selection of five 17-mers (slim red bars) based on the Random Forest prediction as it performed best during cross-validation.

To exemplarily show how the described methods may be used for the prediction of continuous, protective epitopes a recently published relevant epitope on Borrelia burgdorferi OspC has been used [29]. The protein was selected because it was the first to show up in a new IEDB query for functionally altering epitopes and because it shows no significant homology to any protein in the protectivity dataset. Figure 5 indicates the experimentally determined epitope (fat red bar) together with a masked signal peptide (fat gray bar) together with results from three predictive methods. The top-most plot shows the sum-score which does not obviously correlate strongly with the epitope. Even after removal of isolated, positively predicted amino-acids (which can be considered as noise) both the C4.5 prediction and the Random Forest predict amino acids inside the 15-mer epitope. As the Random Forest performed best during validation it is used for the selection of five 17-mer peptides each centered on a cluster of a least two amino-acids. One of these peptides covers more than 50% of the protective epitope. While the proposed methods do not excel on this independently chosen protein five selected peptides may be enough to sufficiently cover the relevant site. As a remark it is also of interest that the consensus prediction of the C4.5 tree and the Random forest would obviously reduce the selection to two peptides with the same epitope coverage.

## Discussion

This work describes the generation of a novel B-cell classifier based on the logistic regression of amino acid parameters derived from principal component analysis of a commonly used parameter database. We extend the classical application of antigenicity classifiers from the prediction of continuous B-cell epitopes to the prediction of protectivity by introducing measured variability and predicted modification patterns into the concept.

We could show that validation on the data-set by Blythe and Flower revealed close to random performance for both the gold standard ABCpred as well as for PCA19, although our method exhibited marginally better performance in comparison. This result is clearly relativated as we could show far better performance on a different dataset when considering only potentially relevant domains.

In detail, to assess the benefit of excluding presumably irrelevant domains from the prediction of protective continuous determinants a manual selection of protein regions was created based on standard bioinformatical tools. For this selection we used a curated set of 57 proteins with known protective or otherwise functionally altering, continuous epitopes. On this compilation protectivity prediction using PCA19 in combination with variability and modification likelihood performed significantly better after domain-accessibility filtering as measured by AROC values, while without filtering performance was comparable (although again slightly better) to antigenicity validation results on the Blythe et.al validation-set. Even when disregading eight proteins which contaminate the validation process due to similarity with the PCA19 training data the difference persists and is even enhanced.

Another primary observation is the gross variance in AROC values observed for various proteins. Approximately 65% of sequences exhibited AROC values < = 0.35 or > = 0.65, indicating a pattern substantially different from random noise. It may be wise to investigate the performance on a per-protein or per-epitope basis and to register how many percent of known, distinct sites were found with high reliability. That may help to avoid an averaging effect leading to the underestimation of the predictive power of classifiers. If a method performs well on every second protein or on certain epitopes that may be sufficient for many practical applications in the life-sciences field. This is especially so where high throughput is involved. Interestingly no correlation between the functional category of a protein or pathogen class (bacterial or viral) and the AROC could be established, indicating a more complex situation than just two types of epitope each associated with a distinct functional class.

By determining the number of theoretically required synthetic peptides to achieve satisfying protectivity in a vaccine or mAb approach we conclude that up to six peptides would be required to achieve success in every second protein. Such success naturally requires the existence of protective, largely conserved and possibly unmodified epitopes.

We also want to point out that the selected validation set presumably represents a combination of differently well mapped proteins. In addition, each of these can contain one or several protective B-cell epitopes of both class I and II. As we tried to detect only one of these in the set, neglecting the other, validation is skewed against our method. It may also be good to remember that calculated AROC values are averaged over entire proteins, not distinct epitopes.

The application of machine-learning procedures potentially combines the prediction of different epitope classes and allows an estimation of the information content in the data. A Random Forest model achieved 83% class separation in an equilibrated model, pointing towards the potential to significantly improve upon the single-score method.

Although the selection of pathogen proteins relevant for the stimulation of protective immune-responses can be enhanced by bioinformatics that is a topic distinct from the prediction of likely protective epitopes on these antigens [30,31]. By combining *in-silico *ranking of likely protective targets and likely protective peptides from these targets completely automatized screening for applicable peptides is possible. Although methods do exist now for both aspects of in silico protectivity screening caution is still necessary. For example, the method published by Doytchinova and Flower predicts 95% of the proteins in our dataset as protective antigens (or at least antigens). On the other hand 65% of the proteome of Staphylococcus aureus COL (1727 of 2618 proteins) are also predicted to be relevant antigens which although possible seems to be a very high number suggesting a higher false positive rate than approximated by validation procedures in the publication. On the other hand all proteins are ranked by a score, so an order of predicted relevance is available, in our view essential for practical use. Unfortunately 701 proteins would be selected to cover all four proteins for which S. aureus protectivity data is available in the IEDB and which have close homologues also in strain COL (fibronectin-binding protein A and B, enterotoxin B and enterotoxin type A), yet not all may be required for a protective immune response and others may contain unmapped or discontinuous epitopes [32]. These findings indicate that several predictive methods and experimental data should be combined when selecting candidates for the generation of protective immune responses.

Our work solely focuses on the prediction of candidate peptides after such a selection has taken place, independent whether by means of experimental data, literature mining or purely bioinformatical/biological considerations.

## Conclusion

We can show that prediction of protective or at least functionally relevant continuous B-cell epitopes can be efficiently done for approximately 50% of 57 analyzed proteins of pathogen origin. By minimizing sequence variability and probability of post-translational modification it can also be assumed that selected peptides are particularly suited for vaccine or monoclonal antibody generation. Exclusion of rationally selected domains strongly enhances the prediction of protective sites, indicating the relevance of a filtering step to restrict to immunologically likely accessible regions. Furthermore, analysis of correlation between variability, conservation of modification profiles and predicted antigenicity shows different and opposed categories of correlation thus indicating the existence of distinct epitope types. At this point it is difficult to verify or falsify our basic assumption of the two-class nature of protective epitopes. On the other hand the high percentage of epitopes with either positive or negative feature correlation indicates the existence of at least two types. Also, decision tree based models significantly outperform the single score-model pointing towards more complex relationships as well as possibly several distinct epitope signatures.

Future paths may lie in the detailed unraveling of parameter-associations and the utilization of more sophisticated classification methods such as regression trees for the assessment of biological relevance and protectivity in the selection of peptides for biotechnological application, as indicated by initial Random Forest classification.

## Methods

### Generation of a B-cell epitope (antigenicity) classifier

The set of 505 unique amino acid propensity scales taken from the AAINDEX database [33] forms a 20 × 505 descriptor matrix of rank 19. Calculation of sequence properties from propensity scales can be expressed as a matrix multiplication of the amino acid sequence in matrix representation with the descriptor matrix. Hence, the resulting property matrix is of rank 19 as well, corresponding to 19 linearly independent vectors. From this only 19 coefficients plus the intercept can be estimated in regression models. Therefore Principal Component Analysis (PCA) was employed to transform the propensity scales from the 505-dimensional space to a 19-dimenstional subspace spanned by all 19 principal components with non-zero Eigenvalues, comparable to how parameter reduction has been done before [34,35]. The 20 × 505 descriptor matrix was centered and scaled to unity variance before the application of PCA. The full information contained in the original 20 × 505 descriptor matrix is retained, because no component is omitted. From this PCA-transformation, a new 20 × 19 descriptor matrix was built. These 19 PCA-derived propensity scales were used in turn to calculate sequence characteristics for the data set. The values were averaged over a sliding window of nine amino acids. Logistic regression employing the logit function was used to build models for the classification of single amino acid residues as epitopic or non epitopic.

In order to estimate the generalization error, bootstrap validation was employed. Error estimates were acquired from out-of-bag validation – i.e. using those residues that are not part of the bootstrap sample as validation data. 50 replicates were calculated for the validation of each investigated model.

### The Blythe and Flower dataset

For validation of antigenicity classifiers a published antigenicity validation set by Blythe et.al (2005) was used. The list of 48 proteins we used from the dataset (because the mapping was clear) can be found in the supplementary material [see Additional file 2]. The data itself can be requested from Blythe et.al.

### Capturing protein variability

To numerically represent variability of a protein at a certain amino acid position an information-entropy measure has been applied. For each protein where variability should be determined the sequence was BLASTed against the non-redundant protein database (nr) and hits were selected manually. The aim was to choose a diverse but not too diverse set of sequences to optimally represent the degree of evolutionary freedom of each amino acid position. Those proteins were downloaded and multiply aligned using clustalw. For each alignment column a variability value was calculated as follows:

Randomly draw 100 samples of size 30 (i.e. 100 times 30 amino acids which is a combination with repetition) from each column, independent of how many sequences have been aligned. That way the evaluation should be less dependent on the number of homologues as for some proteins only five elements can be found whereas for others hundreds are available. Determine the most abundantly found amino-acid in the column. Then calculate the Shannon-entropy weighted by the EMBOSS [36] EBLOSUM variant of the BLOSUM62 [37] substitution scores between each amino acid and the most abundant one in the column. After averaging over all 100 samples to obtain the mean variability the final variability score for each alignment column computes as

$$f(j)=f_{x}log(f_{x})+\sum_{\begin{array}{c} 0<i<\left|j\right|\\ i\neq x \end{array}}^{i\subset j}f(f_{i})=\{\begin{array}{ll} f_{i}log(f_{i})abs(W_{ix}) & \text{if }W_{ix}<0\\ f_{i}log(f_{i}) & \text{if }W_{ix}=0\\ \frac{f_{i}log(f_{i})}{abs(W_{ix})} & \text{if }W_{ix}>0 \end{array}$$

where *f(j) *is the score at alignment column *j*, *f*_*x *_is the frequency of the most abundant letter *x *in column *j*, *f*_*i *_is the frequency of letter *i *in column *j *and *W*_*ix *_is the substitution weight between letters *i *and *x*. For each alignment variability scores are independently rescaled between 0 and 1. The variability score ultimately used for protectivity scoring is *1 – rescaled f(j)*.

Note that each individual gap is regarded as a new character which occurs only once (thus extending the 20 letter alphabet to a potentially high number leading to the perception of strongly gapped positions as highly variable).

As an independent strategy an evolutionary constraint index (ECI) was calculated for each alignment column. This constraint is essentially the difference between the standard Shannon entropy and the entropy after reducing the amino acid alphabet according to the same substitution matrix as above. Briefly, amino acid identities were grouped as follows: E < = E, D, Q, K, R ; I < = I, L, M, V; Y < = Y, W, F, H; S < = S, T, A. Other amino-acids remained ungrouped. The ECI is primarily discussed when parameter correlations are analyzed.

### Posttranslational protein modifications

To predict posttranslational protein modifications PROSITE [38] patterns were used. In particular we considered patterns PS00001, PS00002, PS00003, PS00004, PS00005, PS00006, PS00007, PS00008, PS00009, PS00010, PS00012, PS00013, PS00294, PS00409. All sequences in the previously created multiple alignments were searched for the occurrence of these patterns. For each hit the amino acid putatively carrying the modification was marked and for each column in the alignment the ratio *m *of modified amino-acids was calculated. As for sequence variability the value used for protectivity scoring is *1-m*. The advantage of using motif predictions on aligned sequences is the possibility to derive the degree of conservation of the motif. Combined with the assumption that conserved motifs of post-translational modification are more reliable and do otherwise carry a high false positive rate this increases the weight of the prediction.

### Data for training antigenicity classifiers

A reference data set was generated from the antibody binding site repository BCIPEP [39]. This database holds a collection of experimentally determined B-cell epitopes. The BCIPEP data set is highly redundant with plenty of entries showing relation to more than one source protein.

To realize a non-redundant data set, homologue proteins of this collection were grouped. Members of two different groups differed by at least 30% in sequence identity. After this partitioning, the number of epitopes with length between 6 to 30 amino acids that could be localized on each protein was determined. Finally, the proteins bearing the largest number of epitopes were selected as the representative for their group. This procedure leads to a diverse set of protein sequences with experimentally determined immunogenic regions for each protein.

This data set holds in total 197 proteins with an average length of 449 amino acids. The prevalence of amino acids being part of an epitope is 7.6%. The mean length of an epitope is 16 residues. Out of the 197 proteins 60 originate from bacteria, 55 from viruses, 14 from fungi, 11 from human, 3 from allergens, and 54 from other sources (e.g. eukaryotic parasites). The data set holds in total 401 continuous epitopes, i.e. about 2 epitopes per protein.

Since no systematic epitope mapping of all the proteins in the data set has been performed, categorizing sequence regions as non-antigenic is problematic, as these could be categorized false negative. Epitopes classified as non-epitopes do exist, yet it is problematic to discern whether those are the results of organism or individual (i.e. responses varying from individual to individual) or yet other biases. We chose to declare the non-defined regions of our reference dataset as non-epitopes. Regions not part of an epitope were randomized while maintaining the average amino-acid frequency of BciPep. The resulting was used for training B-cell epitope classifiers.

Each amino-acid functioned as the central amino acid of a 9-mer (peptide), inheriting its class (antigenic or non-antigenic) to the peptide which was then used for parameterization and training/validation. Nine-mers were used due to the desire to use an intermediate between common assumptions about sizes of continuous epitopes (usually between 7 and 15 amino-acids).

### Antigenicity scores for alignments

Each sequence in the generated multiple alignments was independently scored for its antigenicity using the PCA19 classifier. PCA19 classification resulted in the assignment of an antigenicity value for each amino acid in the multiple alignment with the exception of the flanking 5 amino acids due to a window effect. For each alignment column overall antigenicity was calculated as the average antigenicity over the corresponding amino acids of individual sequences.

### Data for validating predictions of protectivity

Proteins with known B-cell determinants were downloaded from the "The Immune Epitope Database and Analysis Resource" (IEDB) [40,41]. The IEDB allows filtering by various criteria. We applied the following step-wise exclusion filters to obtain a protectivity-related dataset:

1. 'Assay Group' = 'Ab binding leading to biological activity' (767 proteins remained)

2. 'Epitope Structure Chemical Type' = 'Peptide/Protein' (735 proteins remained)

3. 'Epitope Source Species' must be defined (679 proteins remained)

4. Exclude linear fragments shorter than 3 and linear fragments longer than 50 aa and structural epitopes (381 proteins remained)

5. 'Qualitative Measurement' = 'Positive' (235 proteins remained)

6. Remove 'Epitope Source Species' = 'Homo Sapiens' (227 proteins remained)

7. Remove identical rows and obvious redundancies or identical sequences in different strains of the same organism. (184 proteins remained).

8. Remove entries from non-pathogens (including pathogenic plants).

SRC6129 and SRC6623 were removed because no Uniprot or GenBank Ids were specified. A neutral protease (gi 30260755) of Bacillus anthracis str. Ames was manually added from a literature source [42]. All proteins were then clustered and identified groups multiply aligned using the standard tools blastclust and clustalw, respectively. Epitopes of all sequences present in the alignment were manually mapped to the homologous sequence where the fewest remapping steps were necessary, or where all epitopes could be represented as can be the case for large deletions or proteins with precursor variants. The process is thus similar to the one applied at the Los Alamos National Laboratory HIV database where all reported epitopes are remapped to the reference strain HXB2 [43].

After removal of all redundancies and impractically short proteins 57 entries (31785 amino acids), with an average peptide coverage (and thus protectivity prevalence) of 7.25% remained, which are from now on referred to as "protectivity dataset". It has to be cautioned that the functional effect of antibodies directed against these determinants is classified only as "leading to biological activity", not necessarily protectivity. For our purposes we consider this close enough an approximation. This dataset can be found in the supplementary materials [see Additional file 1].

### Calculation of validation characteristics and Machine-Learning

Validation results were analyzed using the ROCR package)[44] where specifically AROC (area under the curve of true-positive rate versus false-positive rate plots) calculation has been most relevant.

The WEKA package [45] was used where machine-learning functions were needed, in particular a C4.5 and a Random Forest implementation.

### Synthesis Score

From a practical point of view predictions of continuous epitopes should be measured by the number of synthesized peptides required to cover known epitopes. The Synthesis Score is defined as the number of peptides required to cover at least five epitopic amino-acids in the protectivity validation dataset. Five has been selected as a minimum requirement for an epitope.

### Machine Learning datasets

To analyse the relevance of the used parameters simple machine learning techniques were applied as implemented in the WEKA package. For these analyses a dataset was generated based on the entire protectivity dataset (i.e. not the antigenicity dataset) after exclusion of likely inaccessible regions. Essentially all residues which were likely immune-accessible according to the rules mentioned earlier were represented by the sum score (individually rescaled between 0 and 1 for each protein), antigenicity, PTM pattern conservation and variability. This dataset of dimension 21293 with 1485 antigenic (protective) and 19808 non-antigenic residues (baseline prediction 6.97%) will be termed complete machine learning set (CML set). In a second step for each antigenic (protective) residue a non-antigenic residue was randomly sampled to obtain an equilibrated set. This set was then randomly re-sampled into two stratified sets representing 80% and 20% of CML for training and validation, respectively. The training set (2376 instances) and validation set (594 instances) are termed MTS and MVS, respectively.

## List of abbreviations

ROC – Receiver Operator Characteristic

AROC – Area under the ROC curve

PCA – Principal Component Analysis

PTM – Post-translational modifications

CML – Complete machine learning set

MTS – Machine-learning training set

MVS – Machine-learning validation set

FPrate – False-Positive rate

TPrate – True-Positive rate

Amino-acid Attribute, Parameter – features of an amino acid such as hydrophilicity

C4.5 – A commonly used decision tree algorithm

ECI – Evolutionary Constraint Index

WEKA – The Waikato Environment for Knowledge Analysis

R – The R Project for statistical computing

## Competing interests

The author(s) declare that they have no competing interests.

## Authors' contributions

JS designed the study, assembled the protectivity dataset, performed all analysis based on this data and drafted the manuscript. RG and RR prepared the BCIPEP dataset (removed redundancies) and developed the PCA19 antigenicity classifier. RG, RR, PP and AL worked on the validation of the PCA19 classifier and investigated the contribution of individual sequence features. BM conceived of the study, and participated in its design and coordination.

## Supplementary Material

Additional File 2 **list of protein names for antigenicity validation**. This is a text file listing all proteins from the Blythe et.al. dataset used for the validation of antigenicity. Click here for file

Additional File 1 protectivity validation data. This is a compressed FASTA file which can be viewed (after decompression) using any text editor. Relevant protective or functionally altering epitopes as listed by the IEDB or as determined by homology mapping are contained in the FASTA headers. Click here for file
